# Supplementary material for: Observers of quantum systems cannot agree to disagree
Source: Nat Commun. 2021 Dec 2;12:7021. doi: 10.1038/s41467-021-27134-6 (PMC8640027; doi:10.1038/s41467-021-27134-6)
Supplement: Supplementary file 1 — Supplementary Information [file 41467_2021_27134_MOESM1_ESM.pdf]

Observers of quantum systems cannot agree to disagree. Supplementary Information

Patricia Contreras-Tejada[1], Giannicola Scarpa*[2], Aleksander M. Kubicki[3], Adam Brandenburger[4], and Pierfrancesco La Mura[5]

[1]*Instituto de Ciencias Matemáticas, 28049 Madrid, Spain, patrcont@ucm.es*
[2]*Escuela Técnica Superior de Ingeniería de Sistemas Informáticos, Universidad Politécnica de Madrid, 28031 Madrid, Spain, g.scarpa@upm.es*
[3]*Departamento de Análisis Matemático y Matemática Aplicada, Universidad Complutense de Madrid, 28040 Madrid, Spain, amkubickif@gmail.com*
[4]*Stern School of Business, Tandon School of Engineering, NYU Shanghai, New York University, New York, NY 10012, U.S.A., adam.brandenburger@nyu.edu, http://www.adambrandenburger.com*
[5]*HHL Leipzig Graduate School of Management, 04109 Leipzig, Germany, plamura@hhl.de*

We prove the results in the main text. We also provide the statements and some definitions in the interest of readability.

Supplementary Note 1 - Proof of Theorem 1

Theorem 1. Fix a probability space $(\Omega, \mathcal{E}, \mathsf{P})$ where E_A and E_B are perfectly correlated events. If it is common certainty at a state $\omega^* \in \Omega$ that Alice assigns probability q_A to E_B and Bob assigns probability q_B to E_A, then $q_A = q_B$.

Proof. Since Ω is finite, there is a finite $N \in \mathbb{N}$ such that, for all $n \geq N$, $A_{n+1} = A_n$ and $B_{n+1} = B_n$. From the definition of A_{N+1}, we have that

$$\mathsf{P}(B_N | \mathcal{P}_A(\omega)) = 1 \ \forall \omega \in A_N. \tag{1}$$

Now, A_N is a union of partition elements of $\mathcal{P}_A$, i.e., $A_N = \bigcup_{i \in I} \pi_i$ where each $\pi_i \in \mathcal{P}_A$ and I is a finite index set. From Equation (1), we have

$$\mathsf{P}(B_N | \pi_i) = 1 \ \forall i \in I. \tag{2}$$

*Corresponding author.

Since $\mathsf{P}(B_N|A_N)$ is a convex combination of $\mathsf{P}(B_N|\pi_i)$ for $i \in I$, we must have

$$\mathsf{P}(B_N|A_N) = 1. \tag{3}$$

Now, since $A_N \subseteq A_0$, then $\mathsf{P}(E_B|\pi_i) = q_A$ for all $i \in I$ too. Using a convex combination argument once more, this entails that

$$\mathsf{P}(E_B|A_N) = q_A. \tag{4}$$

Equations (3) and (4) together imply that

$$\mathsf{P}(E_B|A_N \cap B_N) = q_A. \tag{5}$$

But events E_A and E_B are perfectly correlated, so that

$$\mathsf{P}(E_A \cap E_B|A_N \cap B_N) = q_A, \tag{6}$$

as well.

Running the parallel argument with A and B interchanged, we obtain

$$\mathsf{P}(E_A \cap E_B|A_N \cap B_N) = q_B, \tag{7}$$

which implies that $q_A = q_B$. □

Supplementary Note 2 - Relating no-signalling boxes and ontological models

The following result already appeared elsewhere [1]. For convenience, we write down the proof in terms of elementary linear algebra without relying on the sheaf-theoretic language of Ref. [1].

Proposition A1. Given any no-signaling box $\{p(ab|xy) : (a,b,x,y) \in \mathcal{A} \times \mathcal{B} \times \mathcal{X} \times \mathcal{Y}\}$, there is a (non-unique) corresponding ontological model whose probabilities assigned to the states of the world are not necessarily non-negative.

Proof. Let $\{p(ab|xy) : (a,b,x,y) \in \mathcal{A} \times \mathcal{B} \times \mathcal{X} \times \mathcal{Y}\}$ be a no-signaling box. We construct its associated ontological model $\big\{(\Omega, \mathcal{F}, \mathsf{P}),\ \{\mathsf{A}_x^a, \mathsf{B}_y^b : (a,b,x,y) \in \mathcal{A} \times \mathcal{B} \times \mathcal{X} \times \mathcal{Y}\}\big\}$. We provide the proof for $a,b,x,y \in \{0,1\}$ for ease of notation, but the generalization to more inputs and outputs is immediate.

To construct the ontological model, we postulate the existence of a set of states

$$\omega_{a_0 a_1 b_0 b_1} \tag{8}$$

with quasi-probabilities

$$\mathsf{P}_{a_0a_1b_0b_1} \equiv \mathsf{P}(\omega_{a_0a_1b_0b_1}). \tag{9}$$

Each state corresponds to an *instruction set* [2], i.e., the state where Alice outputs a_0 on input $x = 0$ and a_1 on input $x = 1$, and Bob outputs b_0 on input $y = 0$ and b_1 on input $y = 1$. Then, each $\mathsf{P}_{a_0a_1b_0b_1}$ is the quasi-probability of the corresponding instruction set. Of course, if the given box is post-classical, not all of these quasi-probabilities will be non-negative. In fact, in principle it need not even be guaranteed that one can find a quasi-probability distribution over these states. But we will use the probability distribution of the inputs and outputs of the given no-signaling box to derive a linear system of equations over the quasi-probabilities, and show that it does have a solution.

There are 16 states in total, as there are two possible outputs for each of the 4 inputs ($|\mathcal{A}|^{|\mathcal{X}|} \cdot |\mathcal{B}|^{|\mathcal{Y}|}$ in general). Then, each partition corresponds to a set of states as follows:

$$\begin{aligned} \mathsf{A}_x^a &= \{\omega_{a_0a_1b_0b_1} : a_x = a\} \\ \mathsf{B}_y^b &= \{\omega_{a_0a_1b_0b_1} : b_y = b\} \ . \end{aligned} \tag{10}$$

We associate the probabilities $p(ab|xy)$ of the no-signaling box to the probabilities $\mathsf{P}(\mathsf{A}_x^a \cap \mathsf{B}_y^b)$ of each intersection of partitions, for each input pair (x, y) and output pair (a, b). This gives rise to a set of equations for the probabilities $\mathsf{P}_{a_0a_1b_0b_1}$. Indeed, the probability of each intersection is given by

$$\mathsf{P}(\mathsf{A}_x^a \cap \mathsf{B}_y^b) = \sum_{a_{\bar{x}},b_{\bar{y}}} \mathsf{P}_{a_xa_{\bar{x}}b_yb_{\bar{y}}} \tag{11}$$

where we denote the output corresponding to the input that is not x as $a_{\bar{x}}$, and similarly for $b_{\bar{y}}$, and so we have, for each a, b, x, y,

$$\sum_{a_{\bar{x}},b_{\bar{y}}} \mathsf{P}_{a_xa_{\bar{x}}b_yb_{\bar{y}}} = p(ab|xy)\,. \tag{12}$$

Since there are 16 values of $p(ab|xy)$ in the 2-input 2-output no-signaling box, we arrive at 16 equations ($|\mathcal{A}| \times |\mathcal{B}| \times |\mathcal{X}| \times |\mathcal{Y}|$ in general). Of course, there are some linear dependencies between the equations, but we will show that the system still has a solution.

The system of equations can be expressed as

$$MP = C \tag{13}$$

where M is the matrix of coefficients, P is the vector of probabilities $\mathsf{P}_{a_0a_1b_0b_1}$ and C is the vector of independent terms $p(ab|xy)$. The system has a solution (which is not necessarily unique) if and only if

$$\operatorname{rank}(M) = \operatorname{rank}(M|C)\,. \tag{14}$$

Since the rank of a matrix is the number of linearly independent rows, it is trivially true that

$$\mathrm{rank}(M) \leq \mathrm{rank}(M|C)\,, \tag{15}$$

as including the independent terms can only remove some relations of linear dependence, not add more. Equivalently, the number of relations of linear dependence of $M|C$ is always smaller than or equal to the number of relations of linear dependence of M. Therefore, to show that their ranks are equal, it is sufficient to show that every relation of linear dependence that we find in M still holds in $M|C$. That is, for every relation of linear dependence between the probabilities $\mathsf{P}_{a_0a_1b_0b_1}$ that is contained in M, it is sufficient to show that the relation still holds when the sums of probabilities are matched to the elements $p(ab|xy)$ of the no-signaling box in order to show that the system of equations has a solution.

Observe that M contains only zeros and ones, as the equations (12) are just sums of probabilities. Moreover, each column of M corresponds to the probability of a state $\omega_{a_0a_1b_0b_1}$, while each row corresponds to an equation with independent term $p(ab|xy)$. Because the equations (12) correspond to intersections of partitions of the set of $\omega_{a_0a_1b_0b_1}$, we can observe that each row of M has a 1 in the column corresponding to the states $\omega_{a_0a_1b_0b_1}$ contained in the corresponding partition, and a 0 elsewhere. Put another way, in order to construct M one must first partition the set of $\omega_{a_0a_1b_0b_1}$ in four different ways, corresponding to

$$\begin{gathered}\{\mathsf{A}_0^a : a \in \{0,1\}\}\,, \{\mathsf{A}_1^a : a \in \{0,1\}\}\,,\\ \{\mathsf{B}_0^b : b \in \{0,1\}\}\,, \{\mathsf{B}_1^b : b \in \{0,1\}\}\end{gathered} \tag{16}$$

for Alice and Bob respectively. This gives partitions of the columns of M. Then, the 16 possible ways of intersecting partitions of Alice's with partitions of Bob's give the 16 equations with independent term $p(ab|xy)$. But notice now that the partition structure imposes a certain relation of linear dependence between the rows of M. Indeed, for each b, y, we have

$$\left\{\bigcup_a \left(\mathsf{A}_0^a \cap \mathsf{B}_y^b\right)\right\} = \left\{\bigcup_a \left(\mathsf{A}_1^a \cap \mathsf{B}_y^b\right)\right\}\,, \tag{17}$$

as

$$\bigcup_a \left(\mathsf{A}_0^a \cap \mathsf{B}_y^b\right) = \left(\bigcup_a \mathsf{A}_0^a\right) \cap \mathsf{B}_y^b = \Omega \cap \mathsf{B}_y^b = \left(\bigcup_a \mathsf{A}_1^a\right) \cap \mathsf{B}_y^b = \bigcup_a \left(\mathsf{A}_1^a \cap \mathsf{B}_y^b\right)\,, \tag{18}$$

and, similarly, for each a, x we have

$$\left\{\bigcup_b \left(\mathsf{A}_x^a \cap \mathsf{B}_0^b\right)\right\} = \left\{\bigcup_b \left(\mathsf{A}_x^a \cap \mathsf{B}_1^b\right)\right\}\,. \tag{19}$$

Using the correspondence of these partitions with the partitions of the columns of M gives 8 relations of linear dependence between its rows. Now, noticing that a union of columns of M corresponds to a sum of probabilities $\mathsf{P}_{a_0a_1b_0b_1}$, we find that these relations correspond exactly to the no-signaling conditions, as

$$\mathsf{P}\left(\bigcup_a \left(\mathsf{A}_x^a \cap \mathsf{B}_y^b\right)\right) = \sum_a \mathsf{P}\left(\mathsf{A}_x^a \cap \mathsf{B}_y^b\right) \tag{20}$$

so

$$\sum_a \mathsf{P}\left(\mathsf{A}_0^a \cap \mathsf{B}_y^b\right) = \sum_a \mathsf{P}\left(\mathsf{A}_1^a \cap \mathsf{B}_y^b\right) \tag{21}$$

and similarly for Bob. Of course, by definition of no-signaling box, these relations hold for the independent terms $p(ab|xy)$ as well, since

$$\begin{aligned}\sum_a \mathsf{P}\left(\mathsf{A}_x^a \cap \mathsf{B}_y^b\right) &= \sum_a p(ab|xy)\,,\\ \sum_b \mathsf{P}\left(\mathsf{A}_x^a \cap \mathsf{B}_y^b\right) &= \sum_b p(ab|xy)\end{aligned} \tag{22}$$

by construction of the linear system (see equations (11) and (12)). Therefore, every relation of linear dependence between the rows of M holds also between the rows of $M|C$, as required.

Notice also that the implication goes both ways: the linear system has a solution *only if* the set of probabilities $p(ab|xy)$ is no-signaling. The coefficient matrix M incorporates the no-signaling conditions by construction of the states $\omega_{a_0a_1b_0b_1}$ with probabilities $\mathsf{P}_{a_0a_1b_0b_1}$. Therefore, if these conditions do not hold for the independent terms $p(ab|xy)$, then the rank of $M|C$ must be larger than that of M , as $M|C$ contains more linearly independent rows than M. □

Supplementary Note 3 - Classical agreement in no-signalling boxes

We now state and prove the classical agreement theorem in the no-signaling language, i.e. for local boxes. We restrict to boxes of two inputs and two outputs since, by Theorem 5, any larger box exhibiting disagreement can be reduced to a 2-input 2-output box that also exhibits disagreement, while preserving its locality properties.

Recall that a distribution $\{p(ab|xy) : (a,b,x,y) \in \mathcal{A} \times \mathcal{B} \times \mathcal{X} \times \mathcal{Y}\}$ is local if, for each a, b, x, y,

$$p(ab|xy) = \sum_\lambda p_\lambda\, p_A(a|x\lambda)\, p_B(b|y\lambda), \tag{23}$$

for some distributions $\{p_\lambda : \lambda \in \Lambda\}$, $\{p_A(a|x\lambda) : (a,x,\lambda) \in \mathcal{A}\times\mathcal{X}\times\Lambda\}$, $\{p_B(b|y\lambda) : (b,y,\lambda) \in \mathcal{B}\times\mathcal{Y}\times\Lambda\}$ where Λ is an index set.

Theorem 2. Suppose Alice and Bob share a local no-signaling box with underlying probability distribution p. Let $q_A, q_B \in [0,1]$, and let

$$\begin{aligned} p(b=1|a=0,x=0,y=1) &= q_A, \\ p(a=1|b=0,x=1,y=0) &= q_B. \end{aligned} \tag{24}$$

If q_A and q_B are common certainty between the observers, then $q_A = q_B$.

Proof. By definition of q_A, q_B, and using the fact that the shared distribution is local and hence satisfies equation (23), we have

$$\begin{aligned} q_A \sum_\lambda p_\lambda p_A(0|0\lambda) &= \sum_\lambda p_\lambda p_A(0|0\lambda) p_B(1|1\lambda) \\ q_B \sum_\lambda p_\lambda p_B(0|0\lambda) &= \sum_\lambda p_\lambda p_A(1|1\lambda) p_B(0|0\lambda)\,. \end{aligned} \tag{25}$$

From Theorem 3 we have that, if $1 \in \alpha_n$ or $1 \in \beta_n$ for all $n \in \mathbb{N}$ then there is no common certainty of disagreement for any no-signaling distribution, and these encompass local distributions. Hence there only remains to prove the claim for $1 \notin \alpha_n$ and $1 \notin \beta_n$, for some $n \in \mathbb{N}$. This implies that

$$\begin{aligned} p(b=0|a=0,x=0,y=0) &= 1 \\ p(a=0|b=0,x=0,y=0) &= 1 \end{aligned} \tag{26}$$

and hence

$$\begin{aligned} \sum_\lambda p_\lambda p_A(0|0\lambda) &= \sum_\lambda p_\lambda p_A(0|0\lambda) p_B(0|0\lambda) \\ \sum_\lambda p_\lambda p_B(0|0\lambda) &= \sum_\lambda p_\lambda p_A(0|0\lambda) p_B(0|0\lambda), \end{aligned} \tag{27}$$

which implies, on the one hand, that

$$\sum_\lambda p_\lambda p_A(0|0\lambda) = \sum_\lambda p_\lambda p_B(0|0\lambda) \tag{28}$$

and, on the other, that

$$\sum_\lambda p_\lambda p_A(0|0\lambda) p_B(1|0\lambda) = \sum_\lambda p_\lambda p_A(1|0\lambda) p_B(0|0\lambda) = 0, \tag{29}$$

that is,

$$p_A(0|0\lambda) p_B(1|0\lambda) = p_A(1|0\lambda) p_B(0|0\lambda) = 0 \tag{30}$$

for all λ. Therefore, there remains to prove only that

$$\sum_\lambda p_\lambda p_A(0|0\lambda)p_B(1|1\lambda) = \sum_\lambda p_\lambda p_A(1|1\lambda)p_B(0|0\lambda). \tag{31}$$

Because the outputs for inputs $x = 1, y = 1$ are perfectly correlated, we have

$$p_A(0|1\lambda)p_B(1|1\lambda) = p_A(1|1\lambda)p_B(0|1\lambda) = 0 \tag{32}$$

for all λ and, since $p_A(0|1\lambda) + p_A(1|1\lambda) = 1$ and similarly for p_B, this implies

$$p_A(1|1\lambda) = p_B(1|1\lambda). \tag{33}$$

Then we can prove (31) by simple manipulations of the probability distributions of each party:

$$\begin{aligned}
\sum_\lambda p_\lambda p_A(0|0\lambda)p_B(1|1\lambda) &= \sum_\lambda p_\lambda p_A(0|0\lambda)p_A(1|1\lambda)\left(p_B(0|0\lambda) + p_B(1|0\lambda)\right) \\
&= \sum_\lambda p_\lambda p_A(0|0\lambda)p_A(1|1\lambda)p_B(0|0\lambda) \\
&= \sum_\lambda p_\lambda \left(p_A(0|0\lambda) + p_A(1|0\lambda)\right) p_A(1|1\lambda)p_B(0|0\lambda) \\
&= \sum_\lambda p_\lambda \left(p_A(0|1\lambda) + p_A(1|1\lambda)\right) p_A(1|1\lambda)p_B(0|0\lambda) \\
&= \sum_\lambda p_\lambda p_A(1|1\lambda)p_A(1|1\lambda)p_B(0|0\lambda) \\
&= \sum_\lambda p_\lambda p_A(1|1\lambda)p_B(0|0\lambda)
\end{aligned} \tag{34}$$

where we have used the fact that $\sum_{b\in\mathcal{B}} p_B(b|y\lambda) = 1$ for all y, λ in the first equality, (30) in the second and third, $\sum_{a\in\mathcal{A}} p_A(a|x\lambda) = 1$ for all x, λ in the fourth, (32) again in the fifth, and $p_A(1|1\lambda)^2 = p_A(1|1\lambda)$ for all λ (since $p_A(a|x\lambda)$ is either 1 or 0 for every a, x, λ) in the last. □

Supplementary Note 4 - Proof of Theorem 3

In the proof of Theorem 3, we will make use of the following Lemma:

Lemma A2. Consider a no-signaling box of 2 inputs and 2 outputs. Then, $\alpha_0 = \{0, 1\}$ if and only if $q_A = p(b = 1|y = 1)$. Analogously, $\beta_0 = \{0, 1\}$ if and only if $q_B = p(a = 1|x = 1)$.

Proof. We start by proving the direct implication. By hypothesis,

$$\begin{aligned} q_A = p(b=1|a=0,x=0,y=1) &= \frac{p(01|01)}{p(a=0|x=0)} \\ = p(b=1|a=1,x=0,y=1) &= \frac{p(11|01)}{p(a=1|x=0)}. \end{aligned}$$

But now, we can write

$$p(b=1|y=1) = p(01|01) + p(11|01) = p(a=0|x=0)q_A + p(a=1|x=0)q_A = q_A.$$

The reverse implication is trivial. The analogous statement can be proved by interchanging the roles of Alice and Bob. □

Theorem 3. A two-input two-output no-signaling box gives rise to common certainty of disagreement if and only if it takes the form of Supplementary Table 1.

$xy\backslash ab$	00	01	10	11
00	r	0	0	$1-r$
01	$r-s$	s	$-r+t+s$	$1-t-s$
10	$t-u$	u	$r-t+u$	$1-r-u$
11	t	0	0	$1-t$

Supplementary Table 1: Parametrization of two-input two-output no-signaling boxes with common certainty of disagreement. Here, $r, s, t, u \in [0,1]$ are such that all the entries of the box are non-negative, $r > 0$, and $s - u \neq r - t$.

Proof. We first prove that common certainty of disagreement imposes the claimed structure for the no-signaling box. Therefore, we assume common certainty of disagreement, i.e.,

$$(0,0,0,0) \in A_n \cap B_n \qquad \forall n \in \mathbb{N} \tag{35}$$

and

$$q_A \neq q_B. \tag{36}$$

We split the proof into three cases based on the contents of the sets A_n, B_n:

Case 1. $1 \notin \alpha_n$, $1 \notin \beta_n$ for some n.[1] From common certainty of disagreement (equation (35)), we have that

$$p(B_n|a=0,x=0,y=0) = 1, \qquad p(A_n|b=0,x=0,y=0) = 1,$$

[1]This need not happen at the same stage, i.e., possibly $1 \notin \alpha_m$, for some $m < n$. However in this case, since the sets are nonempty by assumption, we have $\alpha_n = \alpha_m$.

which, together with $1 \notin \alpha_n$, $1 \notin \beta_n$, translates into:

$$p(01|00) = 0, \qquad p(10|00) = 0.$$

For q_A, q_B to be well-defined, $p(a = 0|x = 0)$ and $p(b = 0|y = 0)$ must be non-zero, therefore we must have $p(00|00) > 0$.The rest of the table is determined by no-signaling constraints in terms of parameters r, s, t and u. Given the box in the statement of the theorem, $q_A \neq q_B$ if and only if $s - u \neq r - t$, which concludes the proof of this case.

Case 2. $\alpha_n = \{0, 1\}$, for all $n \in \mathbb{N}$ while $1 \notin \beta_m$ for some m. We show that this case implies $q_A = q_B$, so it contradicts common certainty of disagreement. Indeed, the definition of α_{m+1} enforces the conditions:

$$p(b = 0|a = 0, x = 0, y = 0) = 1 = p(b = 0|a = 1, x = 0, y = 0).$$

This implies

$$0 = p(b = 1|a = 0, x = 0, y = 0) = \frac{p(01|00)}{p(a = 0|x = 0)} \quad \Rightarrow \quad p(01|00) = 0,$$
$$0 = p(b = 1|a = 1, x = 0, y = 0) = \frac{p(11|00)}{p(a = 1|x = 0)} \quad \Rightarrow \quad p(11|00) = 0.$$

Adding no-signaling conditions to these last equations, we also obtain

$$0 = p(b = 1|y = 0) = p(01|10) + p(11|10), \tag{37}$$

and so

$$p(01|10) = 0 = p(11|10) \tag{38}$$

and

$$p(b = 0|y = 0) = 1. \tag{39}$$

This allows us to identify q_B with $p(a = 1|x = 1)$, since

$$\begin{aligned} q_B &= p(a = 1|b = 0, x = 1, y = 0) \\ &= \frac{p(10|10)}{p(b = 0|y = 0)} \\ &= p(10|10) \\ &= p(a = 1|x = 1) - p(11|10) \\ &= p(a = 1|x = 1), \end{aligned}$$

where the third and last equalities follow from equations (39) and (38) respectively. Now, taking into account Lemma A2 and perfect correlations, we have

$$q_A = p(b = 1|y = 1) = p(a = 1|x = 1),$$

which shows that $q_A = q_B$, as mentioned above.

Case 3. $\alpha_n = \{0,1\}$, $\beta_n = \{0,1\}$ for all $n \in \mathbb{N}$. We now show that this case also implies $q_A = q_B$, contradicting common certainty of disagreement. Using Lemma A2 we have

$$q_B = p(a = 1|x = 1) \qquad \text{as well as} \qquad q_A = p(b = 1|y = 1).$$

Now, perfect correlations impose that $p(a = 1|x = 1) = p(b = 1|y = 1)$, that is, $q_A = q_B$.

Next, we prove the converse implication of the theorem. We show that any no-signaling box of the above form must exhibit common certainty of disagreement. Since $s-u \neq r-t$, the probabilities Alice and Bob assign to their events of interest are different:

$$\begin{aligned} q_A &:= p(b = 1|a = 0, x = 0, y = 1) = s/r, \\ q_B &:= p(a = 1|b = 0, x = 1, y = 0) = (r - t + u)/r\,. \end{aligned} \tag{40}$$

In the case that $1 \notin \alpha_0$, $1 \notin \beta_0$, we also have that $\alpha_1 = \alpha_0$ and $\beta_1 = \beta_0$, and common certainty of disagreement follows, because $(0, 0, 0, 0)$ is in $A_n \cap B_n$ for all n.

If the parameters are such that

$$\frac{1-t-s}{1-r} = \frac{s}{r}, \tag{41}$$

but

$$\frac{1-r-u}{1-r} \neq \frac{r-t+u}{r}, \tag{42}$$

then

$$p(b = 1|a = 1, x = 0, y = 1) = q_A, \tag{43}$$

as well, but

$$p(a = 1|b = 1, x = 1, y = 0) \neq q_B, \tag{44}$$

and so $1 \in \alpha_0$, $1 \notin \beta_0$. Since we have

$$p(b = 0|a = 0, x = 0, y = 0) = 1, \tag{45}$$

we find $(0, 0, 0, 0) \in A_1$,[2] and hence all A_n still contain $(0, 0, 0, 0)$, yielding common certainty of disagreement.

Symmetric reasoning covers the case $1 \notin \alpha_0$, $1 \in \beta_0$, and only the case where $\alpha_0 = \{0, 1\}$, $\beta_0 = \{0, 1\}$ remains. This happens when

$$\begin{aligned} p(b = 1|a = 1, x = 0, y = 1) &= p(b = 1|a = 0, x = 0, y = 1), \\ p(a = 1|b = 0, x = 1, y = 0) &= p(a = 1|b = 1, x = 1, y = 0) \end{aligned} \tag{46}$$

[2]Note $(1, 0, 0, 0) \notin A_1$, though this does not affect the present proof.

which, in terms of the parameters, is equivalent to

$$\frac{1-t-s}{1-r} = \frac{s}{r}, \tag{47}$$

$$\frac{1-r-u}{1-r} = \frac{r-t+u}{r}. \tag{48}$$

However, these two conditions are satisfied simultaneously only when $s-u = r-t$, as we now show. From Equation (47) we get

$$s = r(1-t),$$

while from Equation (48) we obtain

$$u = t(1-r).$$

This means that if Equations (47) and (48) are both satisfied, then

$$s - u = r(1-t) - t(1-r) = r - t,$$

which contradicts the assumption that $s - u \neq r - t$. □

Theorem 4. No two-input two-output quantum box can give rise to common certainty of disagreement.

Proof. In order to give rise to common certainty of disagreement, the probability distribution that the state and measurements generate must be of the form of Table 1. Theorem 1 in Tsirelson's seminal paper [3] implies that, if there is a quantum realization of the box, then there exist real, unit vectors

$$|w_x\rangle, |v_y\rangle \tag{49}$$

such that the correlations

$$c_{xy} := p(a = b|xy) - p(a \neq b|xy) \tag{50}$$

satisfy

$$c_{xy} = \langle w_x|v_y\rangle \tag{51}$$

for each $x, y \in \{0, 1\}$. For the box in Theorem 3, this means, in particular, that

$$\begin{aligned} \langle w_0|v_0\rangle &= 1\,, \\ \langle w_1|v_1\rangle &= 1\,, \end{aligned} \tag{52}$$

and, since the vectors have unit norm, this implies that

$$\begin{aligned} |w_0\rangle &= |v_0\rangle \ , \\ |w_1\rangle &= |v_1\rangle \ . \end{aligned} \tag{53}$$

Then, we are left with

$$\begin{aligned} c_{01} &= \langle w_0|w_1\rangle \ , \\ c_{10} &= \langle w_1|w_0\rangle \ . \end{aligned} \tag{54}$$

Since the vectors are real, we find

$$c_{01} = c_{10} \, , \tag{55}$$

but this implies that

$$s - u = r - t, \tag{56}$$

which implies that $q_A = q_B$ and, hence, impedes disagreement. □

Supplementary Note 5 - Proof of Theorem 5

Theorem 5. No quantum box can give rise to common certainty of disagreement.

Proof. We show that any no-signaling box with common certainty of disagreement induces a 2-input 2-output no-signaling box with the same property. Thus, if there existed a quantum system that could generate the bigger box, it could also generate the smaller box. Then, Theorem 3 implies that no quantum box can give rise to common certainty of disagreement.

We define a mapping from a distribution $\{p(ab|xy) : a \in \mathcal{A}, b \in \mathcal{B}, x \in \mathcal{X}, y \in \mathcal{Y}\}$ to an 'effective' distribution $\left\{\tilde{p}(\tilde{a}\tilde{b}|\tilde{x}\tilde{y}) : \tilde{a}, \tilde{b}, \tilde{x}, \tilde{y} \in \{0,1\}\right\}$ such that the following conditions hold:

1. if $\{p(ab|xy)\}$ is quantum, then so is $\left\{\tilde{p}(\tilde{a}\tilde{b}|\tilde{x}\tilde{y})\right\}$,
2. if $\{p(ab|xy)\}$ satisfies common certainty of disagreement, then so does $\left\{\tilde{p}(\tilde{a}\tilde{b}|\tilde{x}\tilde{y})\right\}$.

First, notice that the number of inputs can be reduced to 2 without loss of generality, as common certainty of disagreement is always defined to be *at* an event (wlog, $(0,0,0,0)$) *about* another event (wlog, $(1,1,1,1)$). One can associate the inputs $x = 0, y = 0$ with $\tilde{x} = 0, \tilde{y} = 0$, respectively, and $x = 1, y = 1$ with $\tilde{x} = 1, \tilde{y} = 1$ respectively, and ignore all other possible inputs in $\mathcal{X}, \mathcal{Y}$. The outputs, instead,

must be grouped according to whether or not they belong in the sets A_n, B_n (for input 0) and whether or not they correspond to the event obtaining, i.e. whether or not they are equal to 1 (for input 1).

Since p satisfies common certainty of disagreement, we know that $(0,0,0,0) \in A_n \cap B_n$. Moreover, by the definitions of the sets α_n, β_n (and since we only consider finite sets $\mathcal{A}, \mathcal{B}, \mathcal{X}, \mathcal{Y}$) there exists an $N \in \mathbb{N}$ such that $\alpha_n = \alpha_N$ and $\beta_n = \beta_N$ for all $n \geq N$. Take such N, and define the following indicator functions:

$$\begin{aligned} \chi^{\alpha}_{0|0}(a) &= \begin{cases} 0 & a \notin \alpha_N \\ 1 & a \in \alpha_N \end{cases} \\ \chi^{\beta}_{0|0}(b) &= \begin{cases} 0 & b \notin \beta_N \\ 1 & b \in \beta_N \end{cases} \\ \chi^{\alpha}_{0|1}(c) = \chi^{\beta}_{0|1}(c) &= \begin{cases} 0 & c = 1 \\ 1 & c \neq 1 \end{cases} \end{aligned} \tag{57}$$

(where c stands for output a, b for Alice and Bob, respectively), with

$$\begin{aligned} \chi^{\alpha}_{1|x}(a) &= 1 - \chi^{\alpha}_{0|x}(a) \\ \chi^{\beta}_{1|y}(b) &= 1 - \chi^{\beta}_{0|y}(b) \end{aligned} \tag{58}$$

for each a, b, x, y. Then, the mapping from p to $\tilde{p}$ is defined as follows:

$$\tilde{p}(\tilde{a}\tilde{b}|\tilde{x}\tilde{y}) = \sum_{a,b} \delta_{x,\tilde{x}} \delta_{y,\tilde{y}} \chi^{\alpha}_{\tilde{a}|x}(a) \chi^{\beta}_{\tilde{b}|y}(b) p(ab|xy) \tag{59}$$

where

$$\delta_{s,t} = \begin{cases} 0 & s \neq t \\ 1 & s = t\,. \end{cases} \tag{60}$$

We note that the distribution $\tilde{p}$ is obtained from p by means of local pre- and post-processing (see Supplementary Figure 1 for a pictorial representation of the transformation). The classicality and locality of this transformation ensure that $\tilde{p}$ is a well defined non-signalling distribution that is no more non-local than p. In particular, condition 1 above is satisfied.

To check condition 2, i.e. that if $p(ab|xy)$ satisfies common certainty of disagreement, then so does $\tilde{p}(ab|xy)$, let N be as in the definition of the map (59) and let $a \in \alpha_N$. Then, by definition of the set α_{N+1}, we have

$$p(\beta_N|a, x=0, y=0) = 1 \tag{61}$$

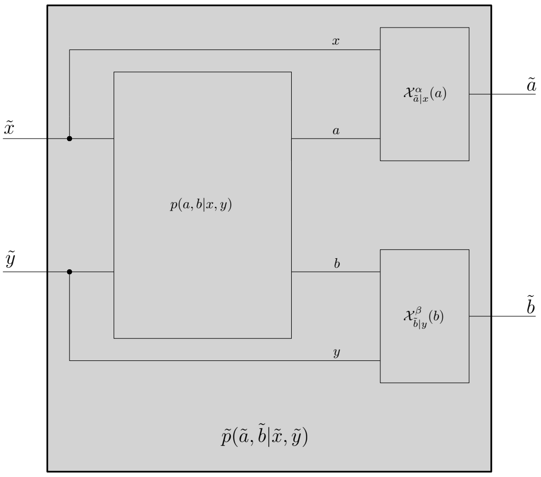

Supplementary Figure 1: A diagrammatic representation of the construction of $\tilde{p}$.

and, therefore,

$$\frac{\sum_{b\in\beta_N} p(ab|00)}{\sum_{b\in\mathcal{B}} p(ab|00)} = 1\,, \tag{62}$$

which entails

$$\sum_{b\notin\beta_N} p(ab|00) = 0\,. \tag{63}$$

Summing over $a \in \alpha_N$, we get

$$\sum_{\substack{a\in\alpha_N\\ b\notin\beta_N}} p(ab|00) = \tilde{p}(01|00) = 0\,. \tag{64}$$

Similarly, we find $\tilde{p}(10|00) = 0$. Since p satisfies common certainty of disagreement, its outputs on input $x = 1, y = 1$ must be perfectly correlated. That is, $p(ab|11) = 0$ if $a \neq b$. Hence,

$$\tilde{p}(01|11) = \sum_{a\neq 1} p(a1|11) = 0 \tag{65}$$

and similarly for $\tilde{p}(10|11)$. So far, the no-signaling box corresponding to $\tilde{p}$ has two zeros in the first row and another two in the last. Using normalization and no-signaling conditions to fill in the rest of the table, we find it is of the form of the no-signaling box in Theorem 3. There remains to check for disagreement, i.e. that if

$$q_A = p(b = 1|a = 0, x = 0, y = 1) \neq p(a = 1|b = 0, x = 1, y = 0) = q_B \tag{66}$$

then

$$\tilde{p}(\tilde{b} = 1|\tilde{a}, \tilde{x} = 0, \tilde{y} = 1) \neq \tilde{p}(\tilde{a} = 1|\tilde{b}, \tilde{x} = 1, \tilde{y} = 0)\,. \tag{67}$$

Since $\alpha_N \subseteq \alpha_0$ and $\beta_N \subseteq \beta_0$, $p(b=1|a^*, x=0, y=1) \neq p(a=1|b^*, x=1, y=0)$ holds in particular for all $a^* \in \alpha_N, b^* \in \beta_N$. This means that, for $a^* \in \alpha_N, b^* \in \beta_N$,

$$\frac{p(a^*1|01)}{\sum_{b\in\mathcal{B}} p(a^*b|01)} \neq \frac{p(1b^*|10)}{\sum_{a\in\mathcal{A}} p(ab^*|10)} \tag{68}$$

and so

$$p(a^*1|01)\sum_{a\in\mathcal{A}} p(ab^*|10) \neq p(1b^*|10)\sum_{b\in\mathcal{B}} p(a^*b|01)\,. \tag{69}$$

Then, we can sum over α_N and β_N on both sides to find

$$\sum_{a^*\in\alpha_N} p(a^*1|01) \sum_{\substack{a\in\mathcal{A}\\ b^*\in\beta_N}} p(ab^*|10) \neq \sum_{b^*\in\beta_N} p(1b^*|10) \sum_{\substack{a^*\in\alpha_N\\ b\in\mathcal{B}}} p(a^*b|01)\,. \tag{70}$$

But in terms of $\tilde{p}$, this corresponds to

$$\tilde{p}(01|01)\sum_{\tilde{a}\in\{0,1\}} \tilde{p}(\tilde{a}0|10) \neq \tilde{p}(10|10)\sum_{\tilde{b}\in\{0,1\}} \tilde{p}(0\tilde{b}|01) \tag{71}$$

which implies

$$\tilde{p}(\tilde{b}=1|\tilde{a}=0, \tilde{x}=0, \tilde{y}=1) \neq \tilde{p}(\tilde{a}=1|\tilde{b}=0, \tilde{x}=1, \tilde{y}=0) \tag{72}$$

and hence the disagreement occurs for the $\tilde{p}$ distribution as well, which proves the result.

Notice that the sets $\tilde{\alpha}_0, \tilde{\beta}_0$ in the distribution $\tilde{p}$ (defined analogously to α_0, β_0 in the distribution p) will correspond to outputs $\tilde{a}, \tilde{b} = 0$, respectively. This is to be expected, as the map $p \to \tilde{p}$ gives rise to a no-signaling box of the form of the one in Theorem 3, where the sets $\tilde{\alpha}_0, \tilde{\beta}_0$ contain a single element each. (In effect, this means we are ignoring the outputs $a^* \in \alpha_0 \backslash \alpha_N$ and $b^* \in \beta_0 \backslash \beta_N$, but those outputs lead to disagreement but not to common certainty of it, so they can be safely discarded.)

□

Supplementary Note 6 - Singular disagreement

Theorem 6. There is no local two-input two-output box that gives rise to singular disagreement.

Proof. Assume Alice and Bob input $x = y = 0$ and obtain $a = b = 0$. This implies

$$p(00|00) > 0. \tag{73}$$

Alice assigns

$$p(b = 1|a = 0, x = 0, y = 1) = 1, \tag{74}$$

and Bob assigns

$$p(a = 1|b = 0, x = 1, y = 0) = 0\,. \tag{75}$$

Further, the outputs for input $(x, y) = (1, 1)$ are perfectly correlated, so, in particular,

$$p(01|11) = 0. \tag{76}$$

Equations (74) and (75) imply, respectively,

$$p(00|01) = 0 \text{ and } p(10|10) = 0. \tag{77}$$

However, equations (73), (76) and (77) make up a form of Hardy's paradox [4], which is known not to hold for local distributions. □

Theorem 7. A two-input two-output no-signaling box gives rise to singular disagreement if and only if it takes the form of Supplementary Table 2.

$xy\backslash ab$	00	01	10	11
00	s	t	$1-s-u-t$	u
01	0	$s+t$	r	$1-s-t-r$
10	$1-u-t$	$u+t+r-1$	0	$1-r$
11	r	0	0	$1-r$

Supplementary Table 2: Parametrization of two-input two-output no-signaling boxes with singular disagreement. Here, r, s, t, u, $\in [0, 1]$ are such that all the entries of the box are non-negative, $s > 0$, and $s + t \neq 0$ and $u + t \neq 1$.

Proof. First, we show that singular disagreement implies that the no-signaling box must be of the above form. By construction, the inputs $x = y = 1$ have perfectly correlated outputs, so that

$$p(01|11) = p(10|11) = 0\,. \tag{78}$$

Also, singular disagreement requires

$$p(b = 1|a = 0, x = 0, y = 1) \;= 1, \tag{79}$$
$$p(a = 1|b = 0, x = 1, y = 0) \;= 0. \tag{80}$$

Equation (79) implies that $p(00|01) = 0$ and $p(01|01) \neq 0$, while Equation (80) implies that $p(10|10) = 0$ and $p(00|10) \neq 0$. The rest of the entries follow from normalization and no-signaling conditions. The condition $s > 0$ ensures that $p(00|00) > 0$, as per the input and output that the observers in fact obtained. Therefore, any two-input two-output no-signaling box that gives rise to singular disagreement must be of the above form.

Proving the converse is straightforward, as it suffices to check that Equations (79) and (80) are satisfied for the parameters of the box. □

Theorem 8. No two-input two-output quantum box can give rise to singular disagreement.

Proof. Ref. [5] guarantees that boxes of Theorem 7 are either local or postquantum. This can be seen by observing that the mapping

$$x \mapsto x \oplus 1\,, \tag{81}$$

which is a symmetry of the box, makes all four 0's lie in entries $p(ab|xy)$ such that $a \oplus b \oplus 1 = xy$. As stated in Sections III and V.B of Ref. [5], all boxes with four 0's in entries of the above form lie in quantum voids. In fact, the boxes in Theorem 7 cannot be local as we have seen that they contain a Hardy paradox, so that all such boxes are postquantum. □

Theorem 9. No quantum box can give rise to singular disagreement.

Proof. Like in Theorem 5, we show that any no-signaling box with singular disagreement induces a 2-input 2-output no-signaling box with the same property, and rely on Theorem 8 to deduce that no quantum system can give rise to singular disagreement.

Analogously to Theorem 5, to prove the Theorem for singular disagreement we define a mapping from a distribution $\{p(ab|xy) : a \in \mathcal{A}, b \in \mathcal{B}, x \in \mathcal{X}, y \in \mathcal{Y}\}$ to an 'effective' distribution $\left\{\tilde{p}(\tilde{a}\tilde{b}|\tilde{x}\tilde{y}) : \tilde{a}, \tilde{b}, \tilde{x}, \tilde{y} \in \{0,1\}\right\}$ such that the following conditions hold:

1. if $\{p(ab|xy)\}$ is quantum, then so is $\left\{\tilde{p}(\tilde{a}\tilde{b}|\tilde{x}\tilde{y})\right\}$,
2. if $\{p(ab|xy)\}$ satisfies singular disagreement, then so does $\left\{\tilde{p}(\tilde{a}\tilde{b}|\tilde{x}\tilde{y})\right\}$.

Again, the number of inputs can be reduced to 2 without loss of generality. To group the outputs, we notice that the sets A_0, B_0 also play a role in singular disagreement, as they group the outputs of each party which lead them to assign their respective probabilities to the event. Then, we group the outputs according

to whether or not they belong in the sets α_0, β_0 (for input 0) and whether or not they correspond to the event obtaining, i.e. whether or not they are equal to 1 (for input 1). We obtain the same mapping (59) as before, substituting α_N for α_0 and β_N for β_0. With this replacement, condition 1 follows by the same proof as before. To check condition 2, we know that, for all $a^* \in \alpha_0$,

$$p(b = 1|a^*, x = 0, y = 1) = 1 \tag{82}$$

and so

$$p(a^*1|01) = \sum_{b \in \mathcal{B}} p(a^*b|01). \tag{83}$$

Summing over $a^* \in \alpha_0$ and rewriting the expression in terms of $\tilde{p}$, we find

$$\tilde{p}(01|01) = \sum_{\tilde{b} \in \{0,1\}} \tilde{p}(0\tilde{b}|01) \tag{84}$$

which implies

$$\tilde{p}(\tilde{b} = 1|\tilde{a} = 0, \tilde{x} = 0, \tilde{y} = 1) = 1. \tag{85}$$

Similarly, for all $b^* \in \beta_0$ we have

$$p(a = 1|b^*, x = 1, y = 0) = 0, \tag{86}$$

hence

$$p(1b^*|10) = 0 \tag{87}$$

and so, by adding over $b^* \in \beta_0$ and mapping to $\tilde{p}$, we find

$$\tilde{p}(10|10) = 0 \tag{88}$$

as required. □

References

[1] S. Abramsky and A. Brandenburger. The sheaf-theoretic structure of non-locality and contextuality. *New Journal of Physics*, 13(11):113036, November 2011.

[2] S. Abramsky and A. Brandenburger. An Operational Interpretation of Negative Probabilities and No-Signalling Models. In F. van Breugel, E. Kashefi, C. Palamidessi, and J. Rutten, editors, *Horizons of the Mind. A Tribute to Prakash Panangaden: Essays Dedicated to Prakash Panangaden on the Occasion of His 60th Birthday*, Lecture Notes in Computer Science, pages 59–75. Springer International Publishing, Cham, 2014.

[3] B.S. Cirel'son. Quantum generalizations of Bell's inequality. *Letters in Mathematical Physics*, 4:93–100, 1980.

[4] L. Hardy. Quantum mechanics, local realistic theories, and Lorentz-invariant realistic theories. *Physical Review Letters*, 68(20):2981–2984, May 1992.

[5] A. Rai, C. Duarte, S. Brito, and R. Chaves. Geometry of the quantum set on no-signaling faces. *Physical Review A*, 99(3):032106, March 2019.
